# COVID-19: a fatal case of acute liver failure associated with SARS-CoV-2 infection in pre-existing liver cirrhosis

**DOI:** 10.1186/s12879-021-06605-7

**Published:** 2021-09-03

**Authors:** Jana Ihlow, Alexander Seelhoff, Victor M. Corman, Achim D. Gruber, Simon Dökel, Jenny Meinhardt, Helena Radbruch, Ernst Späth-Schwalbe, Sefer Elezkurtaj, David Horst, Hermann Herbst

**Affiliations:** 1 Institute of Pathology, Charité–Universitätsmedizin Berlin, Corporate Member of Freie Universität Berlin and Humboldt-Universität zu Berlin, Charitéplatz 1, 10117 Berlin, Germany; 2 Department of Gastroenterology, Vivantes Netzwerk für Gesundheit GmbH Berlin, Vivantes Hospital Spandau, Neue Bergstraße 6, 13585 Berlin, Germany; 3 Institute of Virology, Charité–Universitätsmedizin Berlin, Corporate Member of Freie Universität Berlin and Humboldt-Universität zu Berlin, Charitéplatz 1, 10117 Berlin, Germany; 4 Institute of Veterinary Pathology, Freie Universität Berlin, Robert-von-Ostertag-Straße 15, 14163 Berlin, Germany; 5 Institute of Neuropathology, Charité–Universitätsmedizin Berlin, Corporate Member of Freie Universität Berlin and Humboldt-Universität zu Berlin, Charitéplatz 1, 10117 Berlin, Germany; 6 Department of Hematology, Oncology and Palliative Care, Vivantes Netzwerk für Gesundheit GmbH Berlin, Vivantes Hospital Spandau, Neue Bergstraße 6, 13585 Berlin, Germany; 7 Department of Pathology, Vivantes Netzwerk für Gesundheit GmbH Berlin, Vivantes Hospital Neukölln, Rudower Straße 48, 12351 Berlin, Germany

**Keywords:** COVID-19, SARS-CoV-2, Bile duct, Hepatitis, Case report

## Abstract

**Background:**

The detection of severe acute respiratory syndrome coronavirus (SARS-CoV-2) is challenging, particularly in post-mortem human tissues. However, there is increasing evidence for viral SARS-CoV-2 manifestation in non-respiratory tissues. In this context, it is a current matter of debate, whether SARS-CoV-2 shows hepatotropism.

**Case presentation:**

Here, we report a case of an 88-year-old women with massive SARS-CoV-2 viremia, severe jaundice and clinical signs of an acute hepatitis, who died within a few days from an acute liver failure without showing any clinical signs of pneumonia. Autopsy revealed a severe chronic and acute liver damage with bile duct infestation by SARS-CoV-2 that was accompanied by higher expressions of angiotensin-converting enzyme-2 (ACE2), Cathepsin L and transmembrane serine protease 2 (TMPRSS2).

**Conclusion:**

Our findings indicate an enhanced biliary susceptibility to viral infection with SARS-CoV-2, that might have resulted from pre-existing severe liver damage. Furthermore, our findings emphasize the differential diagnosis of coronavirus disease 2019 (COVID-19)-associated liver failure in the clinical setting of an inexplicable jaundice.

## Background

Over the past year, there have been numerous reports and extensive discussions about the impact of pre-existing health conditions on the clinical course and the severity of COVID-19 [1]. In this context, it is a matter of debate, which cell types except respiratory epithelial cells become infected by SARS-CoV-2 [2] and how this may contribute to the clinical presentation of the disease. Recently, it has been shown that SARS-CoV-2 may be associated with gastrointestinal disorders such as gastroenteritis and hepatitis [3, 4]. However, the evidence on whether SARS-CoV-2 may directly infect, and damage gastrointestinal tissues is scarce [5–7] and should be interpreted with caution. In post-mortem assessment of COVID-19 tissue, the localization of the virus can be hampered by autolysis, enzymatic activity, and pre-existing organ damage. Thus, COVID-19 autopsy results should consequently be interpreted with regard to these limitations which is highlighted by the following case.

## Case presentation

### Jaundice as initial symptom of severe COVID-19

**Fig. 1:**
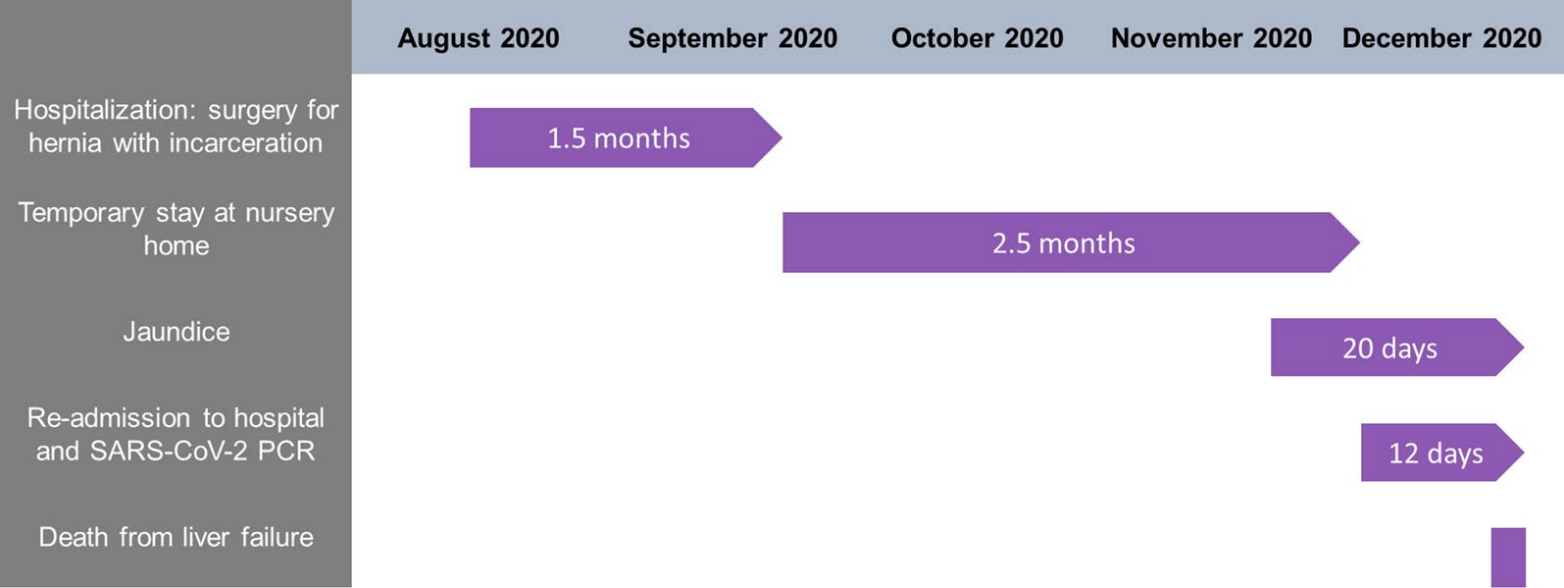
Timeline of events from onset of symptoms to death

Here, we report a case of 88-year-old women who presented with pronounced jaundice and nausea at a care home, 46 days after initial hospital discharge from surgical treatment for an incarcerated femoral hernia with ileus and wound dehiscence. From the medical record, arterial hypertension (treated with Ramipril 5 mg daily), chronic gastritis (treated with Omeprazole 20 mg daily), chronic renal insufficiency (treated with Torasemid 5 mg daily) and a healed pneumonia that had taken place 1 year prior to the events were known as further conditions. During her temporary stay at the care home (Fig. 1) the patient developed a severe jaundice with pale stools. Of note, two other patients were reportedly positive for SARS-CoV-2 infection at the care home.

Due to the rapid deterioration of her jaundice, the patient was re-admitted to the hospital (Vivantes Hospital Spandau, Berlin), where she presented with an unspecific indolent abdominal pressure pain and inactive peristalsis during the physical examination. There were no signs of hepatic encephalopathy or ascites. Neither fever nor respiratory symptoms were present, and the initial SARS-CoV-2 PCR test from a pharyngeal swab was negative. Also, antibodies for SARS-CoV-2 infection were negative in the peripheral blood. However, a second swab, conducted three days later, revealed a positive result. Also, PCR from stool was positive for SARS-CoV-2 RNA. Sampling of the peripheral blood showed a leucocytosis of 15.3/nl and signs of acute inflammation, reflected by a CRP value of 14.5 mg/l, a ferritin level of 7797 µg/l and an interleukin-6 level of 71 ng/l. Furthermore, parameters for cholestasis were excessively elevated above normal levels with a gamma-GT of 397 U/l, an alkaline phosphatase of 269 U/l, and a direct hyperbilirubinemia (direct bilirubin 24.2 mg/dl, indirect bilirubin of 4.5 mg/dl, ratio 5.4). Furthermore, urea levels of up to 78 mg/dl, an LDH level of 253 U/l, an ALT  level of 1,632 U/l, an AST level of 1690 U/l and a high De Ritis ratio of 1.03 reflected a severe liver damage. In addition, parameters of liver function such as coagulation or albumin-synthesis were severely affected with an aPTT of 36.9 s, an INR of 2.12, a quick level (thromboplastin time) of 31% and an albumin level of 29.4 g/l.The GLDH level was 10 U/l, and the ALT + AST /GLDH ratio was 332.2, suggesting a cholestatic genesis. Consecutively, hepatitis A, B, C and E, cytomegaly virus (CMV) associated hepatitis, sclerosing cholangitis (p-ANCA 0.9 U/ml, c-ANCA 2.2. U/ml), primary biliary cirrhosis (ANA negative, AMA IFT 1:320, AMA M2 negative) and autoimmune hepatitis (LKM negative, SMA negative, IgG4 0.197 g/l, IgG 13.44 g/l) were excluded as causes of liver damage. Subsequently, x-ray and CT scans revealed a relapse of the abdominal hernia with a cecal prolapse and a thickening of the intestinal wall. Furthermore, a small scar with pronounced vascular markings was visible in the left lower pulmonary lobe without evidence for pneumonia. The liver, pancreas and spleen presented normal; however, the intrahepatic bile ducts showed a mild dilatation with a diameter of 9 mm in the common bile duct. Abdominal ultrasound and endosonography revealed a thickening of the intra- and extrahepatic bile duct walls and the gall bladder wall without signs of  biliary obstruction. Upon the assumption of a COVID-19 related hepatitis, liver biopsy and convalescent plasma therapy were planned but could not be conducted since the patient’s health condition deteriorated rapidly. Therefore, she could only be treated with best supportive care which  included treatment with L-Ornithine L-Aspartate, Simethicon, Prednisolone, Morphine, antibiotics (Rifaximine, Piperacillin/Tazobactam) and anticoagulative medication (Enoxaparine, Heparin). 12 days after the hospital re-admission, the patient died from acute liver failure. During the entire course of her stay, she had not developed any clinical signs of pneumonia.

### Autopsy and post-mortem diagnostics uncover bile duct infection by SARS-CoV-2

A full body autopsy was performed within 36 h after death, in line with the safety guidelines recommended by the Center for Disease Control and Prevention (CDC) and local ethical guidelines (EA 1/144/13 and EA2/066/20). The macroscopic assessment revealed a subtotal liver dystrophy as immediate cause of death. Furthermore, we found foci which were suspicious of pneumonia in both lower lobes of the lung.

**Fig. 2:**
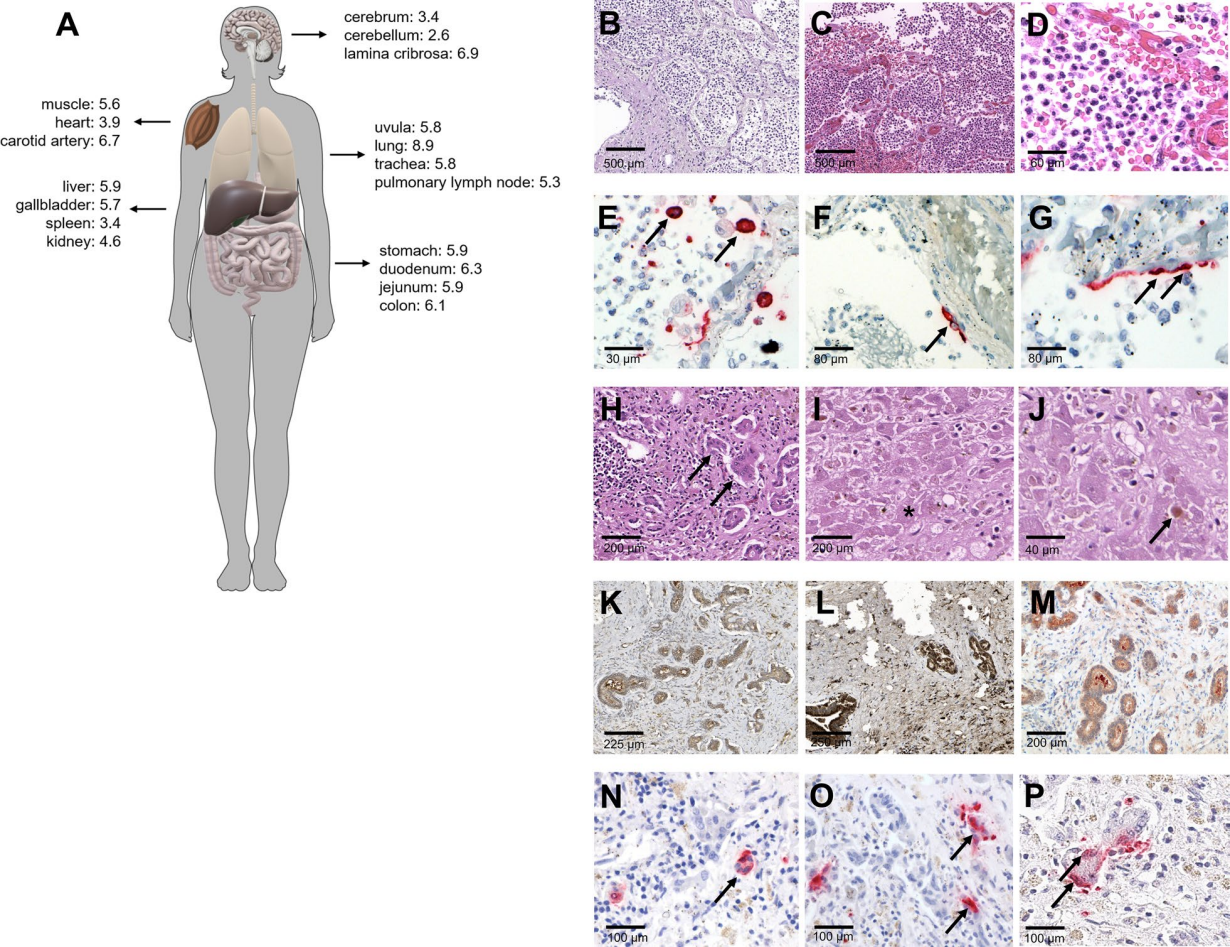
Autopsy results. **A** SARS-CoV-2 RNA copies in each organ system measured as log10 /10.000 cells. **B**–**D** Histopathology of the lung: areas of pneumonic congestion without any further destruction of the lung parenchyma (**B** PAS reaction, **C**, **D** H&E stain). **E**–**G**: SARS-CoV-2 in-situ-hybridization of the lung: specific reaction in pulmonary macrophages (**E**) and pneumocytes (**F**, **G**). **H**–**J** Histopathology of the liver: subtotal liver cirrhosis with moderate portal inflammatory infiltrates and proliferating bile ducts (**H**, arrow), areas of acute liver cell necrosis (**I**, asterisk) and cholestasis (**J**, arrow). **K**–**M** Immunohistochemistry of the liver: strong reactivity of bile duct epithelium for antibodies against ACE2 (**K**), Cathepsin L (**L**) and TMPRSS2 (**M**). **N**–**P** SARS-CoV-2 in-situ-hybridization of the liver: strong cytoplasmatic signals in the bile duct epithelium (arrows)

Native and formaldehyde-fixed paraffin embedded (FFPE) tissue samples were collected from each organ for the assessment of virus burden and histopathology, respectively. Sections of FFPE-tissues were generally stained with hematoxylin–eosin (HE). Additionally, the liver tissue was stained with Periodic-acid Schiff (PAS) reaction as well as Gomori, chromotrope aniline blue and Fouchet stains. Furthermore, we performed immunohistochemical stainings with antibodies for Myeloperoxidase (Dako, polyclonal), CD68 (Dako, #PG-M1), CD20 (Dako, #L26), CD3 (Dako, polyclonal), CD4 (Leica, #4B12), CD8 (Dako, #C8/144B), MUM1 (Zytomed, #MUM1p), ACE 2 (Proteintech, polyclonal), Cathepsin B (Abcam, #ab109131), Cathepsin L (Ptglab, #10938-1-AP) and TMPRSS2(Abcam, #ab109131). Additionally, SARS-CoV-2 in-situ hybridization (ISH) was performed using the ViewRNA™ ISH Tissue Assay Kit (Invitrogen by Thermo Fisher Scientific, Darmstadt, Germany) following the manufacturer’s instructions with minor adjustments. For post-mortem viral SARS-CoV-2 PCR, 50 mg of fresh-frozen samples were acquired (Fig. 2A) and homogenized. Subsequently RNA was purified, using the MagNA Pure 96 system and the MagNA Pure 96 DNA and Viral NA Large Volume Kit (Roche) following the manufacturer’s instructions. RNA extracts were used for quantitative real-time PCR targeting the SARS-CoV-2 E-gene. Viral RNA was quantified by photometrically quantified in vitro RNA transcripts. Total DNA was measured in all extracts using the Qubit dsDNA HS Assay kit (Thermo Fisher Scientific).

Histopathology revealed acute liver cell necrosis with canalicular cholestasis but also a subtotal porto-portal liver cirrhosis with reactive ductular proliferation (Fig. 2H–J) accompanied by a moderate T-cell rich portal inflammatory infiltrate that was partially spreading to the hepatic interface and mainly consisting of CD8 + cytotoxic T-cells (CD8/CD4 ratio 2:1). T-cells accounted for 60% of the infiltrate. In contrast to the controls, the remaining infiltrate was enriched for macrophages (20%), granulocytes (15%) and composed of a small amount of B- and plasma cells (< 3%). Immunohistochemical staining for ACE2, TMPRSS2 and Cathepsin L showed strong membranous signals in the intrahepatic bile duct epithelium (Fig. 2K–M), especially in comparison to the hepatic control tissue. Cathepsin L was strongly expressed in the cytoplasm and on cell membranes of the bile duct epithelium and the hepatocytes both in the patient’s liver and the cirrhotic control but not in the control containing healthy liver tissue. Using SARS-CoV-2 ISH, we found strong signals within the bile duct epithelium , less the possibility of co-existing enzymatic activity (Fig. 2N–P). In contrast, SARS-CoV-2 RNA could not be detected in hepatocytes via ISH. Additionally, post-mortem SARS-CoV-2 PCR confirmed excessive viral RNA load in all tissues with maximum values in the liver, the gallbladder, the gut and the lung. Viral log_10_-SARS-CoV-2 RNA copies are shown in Fig. 2 A. Interestingly, the autopsy also revealed bilateral disseminated foci of pneumonia in the lung and both pneumocytes and alveolar macrophages showed strong signals in the SARS-CoV-2 ISH (Fig. 2), indicating the simultaneous presence of an unrecognized COVID-19 pneumonia despite the lack of clinical symptoms. However, there was no evidence of a diffuse alveolar damage.

## Discussion and conclusions

By revealing liver failure as a foremost symptom of an infection with SARS-CoV-2 even in the clinical absence of pneumonia, this case does not only add further information to the varying clinical presentations of COVID-19 but also underlines the challenge of SARS-CoV-2 localization that complicates COVID-19 post-mortem diagnostics.

Our autopsy results indicate that the 88-year-old woman who presented with inexplicable jaundice was suffering from a previously unknown severe chronic liver damage that was rapidly aggravated by cholestasis following SARS-CoV-2 viremia in the bile ducts, suggesting the clinical picture of a cholestatic subtype of viral hepatitis. However, a direct cause-effect relationship cannot be proven, since the patient also received medication that may have caused hepatotoxicity (such as Piperacilin/Tazobactam or Enoxaparine) and may have accelerated the hepatic decompensation. Moreover, autolysis or enzymatic activity within the bile duct epithelium might have influenced our results. On the assumption, that the combined sensitivity of in-situ-hybridization, immunohistochemistry and SARS-CoV-2 PCR is sufficient, our findings suggest that pre-existing liver damage might have led to a higher susceptibility to SARS-CoV-2 in the bile duct epithelium due to stronger expressions of ACE2, TMPRSS2 and particularly Cathepsin L which has been shown to accelerate cleavage induction and endocytosis of the virus [4]. This theory is supported by similar results found organoids in vitro and via RNA single cell sequencing of hepatic tissue in vivo [8, 9]. Recent studies have also shown that human cholangiocytes are amongst the highest expressors of ACE 2  [10] and that an inflammatory microenvironment, which was obviously present in the previously damaged liver of our patient and potentially triggered by the preceding abdominal conditions, enhances the co-expression of both ACE 2  and TMPRSS2 on hepatocytes and cholangiocytes [4, 11]. Apart from a higher SARS-CoV-2 infection susceptibility in previously damaged tissues, recent studies also promote the theory of an indirect SARS-CoV-2-related end organ damage by activation of the inflammasome [12, 13]. In our patient, this is supported by the enrichment of macrophages, neutrophils, and tissue cytotoxic T-lymphocytes in the inflammatory portal infiltrate. In addition, there is evidence on the hepatotropic potential of SARS-CoV-2 irrespective of previous cell damage [5, 7]. On the contrary, especially electron microscopic evidence should be interpreted with some caution, since cell specific structures that are needed for the procedure are easily damaged by autolysis [14, 15] and diverging results were detected by others with RNA single cell sequencing [16]. Since ISH is more robust (and more likely available in routine diagnostics), we do believe, that our findings are an important and necessary confirmation and addition to the current literature, because they strongly consolidate the theory, that SARS-CoV-2 might infest the biliary epithelium. Since we observed a strong ISH reactivity for SARS-CoV-2 in pulmonary macrophages and high SARS-CoV-2 RNA-values in all mucosal tissues, the possibility of a retrograde bile duct infection cannot be precluded although a direct infection due to higher susceptibility resulting from pre-existing liver damage is far more likely.

Thus, the presented case fuels the general debate on cellular SARS-CoV-2 reservoirs and future studies that implement co-staining and electron microscopy will be necessary to determine the potential direct impact of SARS-CoV-2 on hepatocytes and bile duct epithelium.

## Data Availability

Materials are available from the corresponding author upon reasonable request. However, patient-related data are not available publicly due to ethical restrictions.

## Abbreviations

ACE2: Angiotensin-converting enzyme-2
ALT: Alanine aminotransferase
ANA: Antinuclear antibodies
ANCA (c, p): Anti neutrophil cytoplasmatic antibodies (c = cytoplasmatic, p = perinuclear)
aPTT: Activated partial thromboplastin time
AST: Aspartate aminotransferase
CDC: Center for disease control and prevention
CMV: Cytomegaly virus
COVID-19: Coronavirus Disease 2019
CRP: C-reactive protein
CT: Computed tomography
DNA: Deoxyribonucleic acid
FFPE: Formaldehyde-fixed paraffin embedded
Gamma-GT: Gamma-glutamyltransferase
GFR: Glomerular filtration rate
GLDH: Glutamate dehydrogenase
HE: Hematoxylin–eosin
IFT: Immunofluorescence technique
IgG: Immune globuline G
INR: International normalized ratio
ISH: In-situ hybridization
LDH: Lactate dehydrogenase
LKM: Liver kidney microsomal antibodies
RNA: Ribonucleic acid
PAS: Periodic-acid Schiff
PCR: Polymerase chain reaction
SARS-CoV-2: Severe acute respiratory syndrome coronavirus 2
SMA: Smooth muscle actin antibodies
TMPRSS2: Transmembrane serine protease 2
